# Using a Proximity-Detection Technology to Nudge for Physical Distancing in a Swedish Workplace During the COVID-19 Pandemic: Retrospective Case Study

**DOI:** 10.2196/39570

**Published:** 2022-12-12

**Authors:** My Villius Zetterholm, Lina Nilsson, Päivi Jokela

**Affiliations:** 1 Department of Informatics Faculty of Technology Linnaeus University Kalmar Sweden; 2 eHealth Institute, Department of Medicine and Optometry Faculty of Health and Life Sciences Linnaeus University Kalmar Sweden; 3 Department of Informatics Faculty of Technology Linnaeus University Växjö Sweden

**Keywords:** case study, COVID-19, feasibility, mixed methods, nudging, physical distance, preventive behavior, preventive technologies, proximity detecting technology, wearables

## Abstract

**Background:**

The recent COVID-19 pandemic has contributed to the emergence of several technologies for infectious disease management. Although much focus has been placed on contact-tracing apps, another promising new tactic is proximity tracing, which focuses on health-related behavior and can be used for primary prevention. Underpinned by theories on behavioral design, a proximity-detection system can be devised that provides a user with immediate nudges to maintain physical distance from others. However, the practical feasibility of proximity detection during an infectious disease outbreak has not been sufficiently investigated.

**Objective:**

We aimed to evaluate the feasibility of using a wearable device to nudge for distance and to gather important insights about how functionality and interaction are experienced by users. The results of this study can guide future research and design efforts in this emerging technology.

**Methods:**

In this retrospective case study, a wearable proximity-detection technology was used in a workplace for 6 weeks during the production of a music competition. The purpose of the technology was to nudge users to maintain their physical distance using auditory feedback. We used a mixed methods sequential approach, including interviews (n=8) and a survey (n=30), to compile the experiences of using wearable technology in a real-life setting.

**Results:**

We generated themes from qualitative analysis based on data from interviews and open-text survey responses. The quantitative data were subsequently integrated into these themes: *feasibility* (implementation and acceptance—establishing a shared problem; distance tags in context—strategy, environment, and activities; understanding and learning; and accomplishing the purpose) and *design aspects* (a purposefully annoying device; timing, tone, and proximity; and additional functions).

**Conclusions:**

This empirical study reports on the feasibility of using wearable technology based on proximity detection to nudge individuals to maintain physical distance in the workplace. The technology supports attention to distance, but the usability of this approach is dependent on the context and situation. In certain situations, the audio signal is frustrating, but most users agree that it needs to be annoying to ensure sufficient behavioral adaption. We proposed a dual nudge that involves vibration followed by sound. There are indications that the technology also facilitates learning how to maintain a greater distance from others, and that this behavior can persist beyond the context of technology use. This study demonstrates that the key value of this technology is that it places the user in control and enables immediate action when the distance to others is not maintained. This study provides insights into the emerging field of personal and wearable technologies used for primary prevention during infectious disease outbreaks. Future research is needed to evaluate the preventive effect on transmission and investigate behavioral changes in detail and in relation to different forms of feedback.

## Introduction

### Background

Since the onset of the COVID-19 pandemic, governments across the globe have issued a range of different mitigation and control measures to reduce transmission. At the individual level, these measures have included, for example, promotion of hygiene routines, wearing a face mask, various levels of contact tracing, lockdowns, and restrictions in movement and crowding in an effort to maintain physical distance between individuals [1]. Consequently, there has been a worldwide interest in using wearable and mobile information technologies for preventive work [2]. Gasser et al [3] provided a typology of digital tools against COVID-19; these include 4 categories of technologies, namely *flow modeling*, *quarantine control*, *symptom monitoring,* and *proximity and contact tracing*. The primary interest of this study is in the fourth category, comprising technologies for digital proximity tracing.

Contact-tracing apps (CTAs) have been one of the most frequently implemented technologies in this vein [4-7]. According to most conceptualizations, digital proximity tracing is synonymous with digital contact tracing; that is, it is implied that this technology can only be used for this purpose in a pandemic situation. This perception is exemplified in the typology [3] mentioned earlier as well as in definitions of proximity-tracing solutions provided by the European Centre for Disease Prevention and Control and the World Health Organization, the latter of which says, “Digital proximity tracing refers to a technological approach to public health contact tracing that typically utilizes smartphones or purpose-built devices to capture anonymized interactions between individuals, and subsequently issue alerts, if conditions are met that indicate a period of close proximity to someone who later returns a positive diagnosis of infectious disease” [8].

CTAs are a digital version of the classical manual contact-tracing method commonly used during infectious disease outbreaks. The implementation of CTAs can be voluntary or mandatory. The design of these apps varies in different countries [6] and so does their uptake and acceptance in different populations [7,9-11]. The deployment of contact-tracing technologies has raised many concerns regarding ethics, privacy, and feasibility [12-16].

In contrast to the standard perception of proximity and contact tracing, some studies have suggested that proximity-based technologies can be used for purposes other than contact tracing to support users’ situational awareness. For example, they could be used to alert users with a nudge or similar notification, providing them with immediate feedback of their physical distance to another individual [17-19]. As such, this approach exemplifies a design thinking approach to the use of wearable technologies for infectious disease management, where new areas of application are explored based on existing technologies. This approach emphasizes primary prevention by deploying proximity technologies to achieve a direct impact on behavior and thereby placing the individual user in control of the situation. This differs from the traditional focus of CTAs, which involves the tracking of infectious individuals. However, the practical feasibility of proximity tracing for nudging purposes has not been well studied in the context of infectious diseases. To date, most studies have been theoretical [17,18] or have investigated the effectiveness of this technology in experimental settings [19]. This approach needs to be further studied and include real-world aspects such as behavioral outcomes and user experience [17,19].

### Designing Preventive Behavior

A pandemic is a complex system of interacting factors that need to be understood from biological, social, and psychological perspectives, to name a few. A root cause of transmission is the behavior of individuals [20], that is, the human hosts. Therefore, the promotion of preventive behavior is a key aspect in any infectious disease outbreak, and there is a need for evidence-based strategies for the intervention and formation of new patterns of behavior to limit transmission. In the early days of the current pandemic, models and methods from behavioral science were discussed to inform the development of preventive interventions [20-22].

The importance of protective and preventive behavior has been evident in much of the public communication during the COVID-19 pandemic, and simple strategies such as hand washing, wearing a mask, and social and physical distancing have been repeatedly promoted around the world [23,24]. The appropriate use of preventive measures depends on the availability of personal protective equipment, the type of activities, and the physical context. In an experimental study conducted in a laboratory setting, a model system of dolomite dust particles was used to simulate airborne transmission [25]. It was shown that if everyone properly wears a highly efficient and tight-fitting mask, such as FFP2 or KN95, the risk of airborne transmission is very low. The risk increases if only the susceptible person is wearing the FFP2 mask, if surgical masks are used, and if physical distancing is maintained without masking [25]. However, uncertainties remain regarding the significance of various transmission routes [26]. In a retrospective case-control study in community settings in Thailand, it was found that consistent mask wearing, handwashing, and adhering to social distancing were all independently associated with a lower risk of infection, but none of these protective measures alone could provide complete protection from the infection. Complying with all measures was the most effective way to reduce transmission in public gatherings, and it was also found that those who consistently and correctly wore masks were more likely to wash their hands and practice adequate social distancing [27].

However, adhering to preventive recommendations can be challenging. New behaviors will require a change in habit, and cognitive factors such as attention span or memory may prevent full adherence to public health recommendations. Even with information and knowledge, changing subconscious routines or automatic behaviors is challenging [24]. Furthermore, there have been few opportunities for training or support intended to ameliorate these problems or help form new habits [20]. A few novel technologies have emerged that can promote and support new preventive habits such as adherence to handwashing routines [28]. It has also been suggested to add more persuasive design elements to the existing CTAs to increase users’ uptake and adherence to public health recommendations [29]. As previously mentioned, technologies that could support physical distancing by increasing users’ situation awareness have also been proposed [17-19].

The design of warning systems aimed at improving safety-related behaviors has been studied in a range of contexts. Proximity-based warning systems are often used in dangerous working environments where there is a heightened risk of collisions between people and heavy equipment, such as construction sites, manufacturing plants, and underground mines [30-32]. They have also been used in surgery rooms where extreme attention to details is needed [33,34]. Warning systems in general are also common in various driving assistance systems and semiautonomous vehicles [35-37]. A common feature that has been studied in these various environments is the effectiveness of visual [32], auditory [30], and vibro-tactile [31] signals and multimodal combinations [33-37] of these alerts. Research from dangerous working environments has shown that even with safety standards and planning, workers often fail to recognize or act on safety hazards owing to factors such as lack of attention, cognitive overload, and distractions [30]. These observations have led to an increasing interest in how technologies can help workers recognize and avoid hazards. However, studies on these types of systems also indicate that repeated exposure to warnings may desensitize the user and cause alarm fatigue, particularly if alerts are too frequent, impossible to avoid, or redundant. Recent research efforts have aimed to minimize these types of issues [30,31].

An increasingly popular approach to address cognitive limitations, such as a lack of attention, is nudging. In this study, the term *nudging* [38] refers to an approach that supports behavior change by design rather than behavior change as a result of intention and attitude change. Nudges are small and purposeful design elements that can take the form of, for example, reminders and notifications [38]. A nudge can be defined as “a function of the choice architecture that alters people’s behavior in a predictable way that is called for because of cognitive boundaries, biases, routines, and habits in individual and social decision-making and which works by making use of those boundaries, biases, routines, and habits as integral parts of the choice architecture” [39].

A main goal of nudging is to design in a way that is expected to encourage individuals to act in their own best interest [38,40]. The design focus of nudging lies in purposeful changes to the choice architecture, that is, the context in which people act and where the designer can implement nudges. One principle for designing nudges is to provide feedback that can support individuals by informing them when they make mistakes [38]. This type of nudge can be defined as just-in-time prompts that draw attention to a behavior when it occurs [41]. Therefore, nudges are particularly relevant in situations in which the main goal of the design is to approach the automatic behavior of individuals. In recent years, there has been a heightened interest in using various information technologies to nudge users, which has influenced design approaches in both information systems and human-computer interactions [41].

Nudges can be classified based on multiple perspectives. One model categorizes whether they focus on the reflective or automatic mind, whether they target behavior or choice, and the level of transparency versus nontransparency in the design of nudges [42]. The technology described in this study provides a nudge that would be categorized as focused on the automatic mind and targeting behavior and is transparent for the user. The physical form of the device, called the distance tag, was a credit card–sized wearable that hung around the necks of individuals when they were in the workplace. If the individuals wearing these tags came within 1.5 m of each other, they were alerted by a high-pitched audio signal. This means that the nudge was designed as a just-in-time prompt and provided the user with audio-based feedback on mistakes. It should also be noted that the distance tag could be switched off.

### Objective

There has been little research on nudging technologies in the context of infectious disease. The case described in this study is an early example of a large-scale workplace setting in which an emerging nudging technology was used to achieve primary prevention during a pandemic. The technology used on-site, that is, the distance tag, incorporated behavior change by design to improve physical distancing and limit transmission. The main objective of this study was to evaluate the feasibility of using a wearable device to nudge for distance and to gather important insights about how functionality and interaction were experienced by the users. Another goal of this study was to inform future design efforts in this field. The following research questions guided this study:

1. How feasible is it to use proximity-based technology to nudge users for physical distance in a large-scale workplace setting?
2. How did users experience functionality and interaction with this technology?

This study is a retrospective case study [43] that assessed the feasibility and experience of using a distance tag in a workplace for 6 weeks during the production of a television-broadcasted music competition (also referred to as *the production* or *production project* in this paper). We were not involved in the production, design of the preventive strategy, or the implementation of the distance tag. The setting of the case offered the unique possibility to learn more about whether wearable proximity technologies can be used in natural settings where individuals meet, work, and socialize during an infectious outbreak. The mixed methods approach in this study contributes with 2 complementary perspectives. The interview participants were those who were responsible for management and safety during the production; therefore, they could provide a holistic perspective of the case. The survey participants contributed with their individual experience and perception of the technology. The results of this study can contribute to knowledge about the use of preventive and proximity-based technologies and can guide further design efforts in this emerging field.

## Methods

### Overview

Because the COVID-19 pandemic and technologies used here are both novel, this study was conducted using an explorative approach. This was a retrospective case study [43], in which the research study and all data collection were conducted after the production activities were completed and the music competition was broadcast. The researchers collected the data based on the experiences of the participants.

The general rationale of a mixed methods study design is that a combination of both qualitative and quantitative approaches provides a better understanding of research problems than only 1 approach [44]. This study had an exploratory sequential design [44], where the first inquiry included qualitative interviews, the results of which informed the second inquiry, that is, the development of the survey and quantitative data collection. This sequential procedure is an example of instrument development [45]. The final analysis combined results from both qualitative and quantitative studies to gain a more comprehensive account of the studied phenomenon, which can be referred to as completeness [45]. The overall study design is presented in Table 1.

**Table 1:** The mixed methods design used in this study.

Step	Approach	Method	Time	Sampling technique and sample size
1	Qualitative	Preliminary unstructured interview	February 2021	Purposeful sampling (n=1^a^)
2	Qualitative	Semistructured individual interviews (interview A+B)	March 2021	Snowball sampling (n=2)
3	Qualitative	Semistructured group interview (interview C)	March 2021	Snowball sampling (n=6)
4	Mixed methods quantitative	Web-based questionnaire	April 2021	Total population sampling (n=30)
5	Mixed methods	Final analysis of results from both qualitative and quantitative study	October- December 2021	8 qualitative+30 quantitative (n=8+30; total n=38)

^a^This individual was later included in a more formal group interview (step 3, interview C).

### Interviews

The first contact was an informal talk (unstructured interview) in which the researcher contacted the person responsible for the preventive strategy of the production project. The informant described some of the key aspects of the case and their collective experiences, which provided the researcher with initial insight into the case and contributed to the topic guide in the forthcoming interviews. These topics included expectations of the distance tag, how information about the distance tag was distributed, and how the distance tag worked in the setting of the production. The following phase included semistructured interviews with 8 individuals. Interview candidates were selected and contacted using a snowball approach, in which the first respondents suggested others who had insights into the preventive strategy. The participants were strategically selected [46] based on their position in the production project, their experiences from working in the studied setting, and their engagement with the technologies in focus. The selected interviewees had worked at the site of production for 6 weeks and had experience using and helping others to use the distance technology. These interviewees had management roles, technological roles, or safety-related roles in the production project and therefore had a good overview of the perspectives of both participants and management. Thus, they could provide an overview and a holistic perspective on the role of this preventive technology during the entire production process. Two individual interviews and 1 group interview with 6 individuals were conducted in March and April 2021 (interviews A, B, and C) using a videoconferencing software. All interviews were conducted in Swedish; direct quotes from the interviews were translated by the authors and proofread by a translation service.

### Survey

On the basis of the findings of the interviews, a questionnaire was developed to survey the individual experiences of the distance tag. It was based on the key topics and relevant issues from the interviews. The purpose was to reach a larger set of users and thereby gain a broader perspective on experiences. The questionnaire (referred to as the survey) was offered to the participants in Swedish, and the translation of the original text is available in Multimedia Appendix 1. The survey included both closed questions (multiple choice with a set of alternatives or a rating scale) and open-ended questions (text-based responses), the latter of which provided opportunities for participants to expand the answers beyond the set alternatives and to motivate and explain their choices. The exploratory sequential approach and the inclusion of open-ended responses were motivated by the nature of the rapidly evolving pandemic situation and the emerging field of technologies that is being studied in this case. To our knowledge, no preexisting theory or assessment scale has been developed for this specific context. After this 6-week production had ended, a link to the survey was shared via an app used in the production project. A reminder email with a link to the survey was sent so that the survey would be available for everyone who had been involved in the music competition and production (approximately 400 individuals). A total of 30 individuals responded to the questionnaire. A time delay of approximately 2 weeks between the production project ending and the first distribution of the survey may have affected the response rate because by that time, many of the participants may not have been actively staying up to date with information in the project. Participation in interviews and the survey was voluntary, informed consent was collected, and no personal health data were collected during this study.

### Data Analysis

The interview responses were analyzed using thematic analysis. The approach was inductive, as the themes were generated based on the collected data, that is, the users’ experiences of a technological approach to support the maintenance of physical distance in their workplace. The coding and theme generation were descriptive and conducted on a semantic level; that is, based on the interviewees’ explicit content, no assumptions were made regarding the latent underpinnings of the data [47].

We read the transcribed interviews several times. We highlighted parts of the text that focused on the aim and research questions. The highlighted parts of the text were then condensed into 1 or 2 sentences, and sentences were grouped into categories. As a final step in the qualitative analysis, key topics were identified and used as a basis for quantitative data collection through the questionnaire. The open-ended responses were analyzed based on their semantic content and combined with other results in the final stage. The first subthemes were generated from the qualitative analysis based on data from interviews and open-text responses in the survey, after which the subthemes were merged to create 2 main themes. The quantitative data were subsequently integrated into these themes.

The quantitative data from the survey primarily consisted of categorical choices and ordinal Likert-type scales, survey data of the closed questions are available in Multimedia Appendix 2. The results are presented using descriptive statistics, mainly frequency distributions and the mean or median for the central tendency. A potential change in attitudes about using distance tags before and after the 6-week production project was tested using the Wilcoxon signed-rank test, which is a nonparametric test to account for different measurements of the same individuals for ordinal scale data [48]. The null hypothesis was that there would be no significant difference between attitudes before and after using the distance tag in the workplace.

### Ethical Considerations

On the basis of the general guidelines of the Swedish Ethical Review Authority, this study was not classified under the requirements for ethics approval in Sweden [49]. This was a retrospective case study, meaning that we as researchers were not involved in implementing the technology, and the research study began at the end of the music competition to compile the learnings from this case. The respondents were asked to describe their experiences with the technology. From an ethics standpoint, this was an observational study. Furthermore, the study did not set up individual registers or collect personal identifiable information or health data nor did it collect information about religion, sexual orientation, or political preferences. Information about the total number of infected cases was summative and disclosed by the management; no such data were collected from the study participants, nor could those cases be connected to any specific individual.

All participation was voluntary, requiring opt-in to consent, and respondents could opt out at any time. Before the interviews, the participants were informed about the study both orally and in writing. The survey participants were informed and asked to participate in writing. The informed consent form and oral information were provided in Swedish. The information included the purpose of the study, how the research was to be conducted and reported, and how the collected data were to be stored; the fact that participation was voluntary; the expected duration of the participation; and expected benefits and foreseeable discomforts to the prospective participants. The information provided also stated that the survey respondents were anonymous, and the identities of the interviewees were confidential.

Before the interview began, the participants were asked orally if they had read the information, if they had any questions, and if they consented to participate. The survey participants’ consent was ensured by an information text at the end of the survey, stating that if they chose to send the survey, they agreed that their answers could be included in the study. No compensation was offered to participants.

## Results

### The Case Background

This case involved the production of a publicly funded music competition that took place in Sweden in February and March, lasting for 6 weeks in 2021. In addition to artists, production team members, and technical staff, this yearly competition usually attracts large live audiences, but owing to the pandemic, there was no large live audience in this particular year. Challenges remained because of the large number of people involved in the music competition and production (approximately 400 individuals). To be able to go through with the production and ensure the best possible safety for the participants, a substantial preventive strategy was planned based on national public health recommendations. The preventive strategy included stations for handwashing, forming “social bubbles” with allocated restrooms, measures to ensure physical distancing, and, when the appropriate distance could not be maintained, the use of face masks.

Physical distancing (≥1.5 m) was among the most important recommendations in Sweden, and therefore a key issue was finding a way to support distancing behavior and ensure that distance was maintained throughout the workday and production project. The production’s COVID-19 strategist decided to test a proximity-based wearable technology that continuously measured the physical distance between individuals. The function is that, if the wearables come within 1.5 m of each other (ie, the individuals wearing them are not keeping their distance), an alarm is tripped and the wearer is notified. Occasionally, the distance tag could be switched off when the sound is inappropriate, for example, during recording sessions.

A pilot test was conducted using a Bluetooth-based device, but the proximity-based detection was not sufficiently specific, so this technology was deemed to be unfeasible for this production and workplace. A new type of device based on an ultrawide band (UWB), called a distance tag, performed better in proximity detection, so this technology was implemented. The physical form of the device was a card that hung around the necks of individuals entering the facilities. The proximity detection was set to 150 cm, and as the detection error margin was 20 cm, the alarm sounded when the distance between 2 persons was approximately 130 cm to 170 cm. Everyone who entered the facility was strongly advised to wear a distance tag. The device also has a mobile app with more functionalities and an option to enable tracking for contact tracing, but this app was not used during the event because of privacy concerns. In this setup, the UWB device enabled a direct reminder (nudge) for distance but no follow-up or tracking functions.

The total number of participants in the interviews was 8 (ie, 5 men and 3 women); the number of participants in the questionnaire was 30 (ie, 18 men and 12 women). The interviewees were responsible for the production, personnel, or security on-site, whereas most of the questionnaire participants worked in the production. The age distribution of the participants was between 28 and 68 (mean 46.2, SD 1.96) years.

In the analysis, 2 main themes and 7 subthemes were identified: feasibility (*implementation and acceptance—establishing a shared problem*; *distance tags in context—strategy, environment, and activities*; *understanding and learning*; and *accomplishing the purpose*) and design aspects (*a purposefully annoying device*; *timing, tone, and proximity*; and *additional functions).*

### Feasibility

In this main theme, the focus is on the use of a wearable device to nudge for distance. Four subthemes are described in this section: *implementation and acceptance—establishing a shared problem*; *distance tags in context—strategy, environment, and activities*; *understanding and learning*; and *accomplishing the purpose*.

#### Implementation and Acceptance—Establishing a Shared Problem

The participants received information about the preventive plan before the distance tags were implemented. Some participants were curious about the technology, while others were skeptical. Several survey respondents noted that their participation was motivated by a desire to prevent infection in themselves and others:

The motivation to prevent transmission during the production as much as possible.

Survey respondent

The others were not initially assured that this type of device was needed. Several interviewees said that not everyone considered keeping distance to be a problem; that is, they thought that they were already good at maintaining sufficient distance from others. However, using proximity technology to continuously monitor behavior made it clear that this was not the case. Several interviewees described that the device helped them be attentive to distance and taught them how much distance they needed to maintain:

Distance can be challenging. When you start using the tags, you understand more “how much distance.” And you understand that what you had before might not have been a [sufficient] distance...

Interview C

I’ve heard about “keep the distance.” And of course, we have structured our work to avoid getting too close. But I don’t think I understood how...That you were close until I’ve tried these...I mean, I really got to learn what a good distance is, so even if I believed that I had it before, I have, well, it has opened my eyes for it.

Interview A

Other interviewees described that when these kinds of devices are implemented, clear and effective communication is important so that the users understand why maintaining a safe distance might be difficult:

Our perception is that we have created the conditions so that people can maintain a distance. But at the end of the day, this is an individual responsibility.

Interview C

The interviewees also described some practical lessons learned from the implementation. At first, they started by handing out distance tags to everyone and then let users be responsible for their devices. Users were supposed to take the tag home, charge the batteries, and bring it back to the workplace every day. However, this approach did not work well because users forgot their tags at home or forgot to charge the batteries. Instead, a station was set up at the entrance so that distance tags and battery checks were available for everyone entering the site. This step improved the process and made it easier to check that the devices worked and that everyone had a device.

From the managerial perspective, the impression of acceptance was that most people accepted the distance tag sooner or later, even though the feelings were mixed at the beginning. Some felt that the distance tags were slightly disruptive and put them away or switched them off regularly. However, this situation improved after an informational campaign that focused on preventive measures, including information about the need for both face masks and distance tags, asking people questions (self-assessment), and controlling whether the devices were active. From the managerial perspective, the preventive measures, including using technological support to maintain physical distance, were ways to ensure everyone’s safety so that the production project and music competition could be carried out. Security personnel noted that users perceived these measures as a means of protecting their health. After more information was conveyed to users that the preventive measures were meant to protect not only themselves but others as well, the impression was that both adherence and acceptance increased. The interviewees’ perception was that people needed time to get used to the devices but had respect for the situation:

People were a bit unprepared, but acceptance grew over time.

Interview A

People understood that we need this to be able to go through with an event like this during a pandemic.

Interview B

The interviewees described another benefit of the devices, which was that the alarm could be a way to avoid conflict or feeling embarrassed about having to ask someone to step back. From the perspective of security personnel, this aspect also improved their working environment, because they did not have to interrupt people in the midst of a conversation to tell them to move back.

In the questionnaire, the participants were asked about their attitudes toward using distance tags, this included to estimate how they felt both before and after the production project. The response option in both questions was a 5-point rating scale (Table 2).

The descriptive statistics show that after the production project, there were no negative responses, the number of neutral responses decreased, and both skeptical and positive responses increased. The median value before and after distribution was *quite positive* (rating 4). The difference between attitudes before and after the intervention was not statistically significant (*W*=108; *P*=.80), so the null hypothesis cannot be rejected.

The overall experience was measured using the questionnaire with 2 questions, where the response was given on a 5-point rating scale (Table 3).

Table 3 shows that the overall experience was mainly positive as the majority of answers (47/60, 78%) were *agree* or *strongly agree*.

**Table 2:** Response distribution of attitudes toward using distance tags before and after the production project (N=30).

Rating	Response	Before	After
1	Negative—the tags will not work well or did not work well	1	0
2	Skeptical	5	8
3	Neutral—do not know	5	1
4	Quite positive	9	9
5	Positive—the tags will work well or worked well	10	12

**Table 3:** Overall experience of using the distance tags.

Questions	Strongly disagree	Disagree	Neutral	Agree	Strongly agree
As a whole, I support this effort	0	3	3	14	10
I’d recommend the tag for other workplaces	0	3	4	11	12
Total	0	6	7	25	22

#### Distance Tags in Context—Strategy, Environment, and Activities

Distance tags were embedded in a larger battery of measures as part of the overall preventive strategy. The connection between the distance tags and the preventive context was a key issue for their feasibility. This holistic approach involved a combination of measures. For instance, face masks were used in situations when distance could not be maintained. In addition to face masks, other preventive measures included handwashing stations, fever scanning at the entrance, and the formation of social bubbles. The latter meant that people working closely together at the event also shared hygiene facilities. The interviewees further described that the combination of distance tags, personal protective equipment, handwashing stations, effective communication, and people overseeing the adherence to these measures was necessary for a successful preventive strategy:

Of course, there are occasions when it won’t work, in this kind of production. In these cases, we have other measures.

Interview A

It was good to continuously get a reminder of the distance, it made you more meticulous with other things such as washing your hands and using face mask.

Survey respondent

The interviewees emphasized the importance of the event’s venue as being sufficiently spacious for individuals to maintain physical distance. One of the key aspects for the successful use of distance tags is that the tags fit in the physical environment. They are particularly useful in places and situations where people meet and tend to stand close to each other or where people’s paths cross, for example, by coffee machines. Manual adjustments regarding proximity detection to fit the conditions of the event could be made, but there were situations in which the distance tags were not deemed useful. Some survey respondents commented that the tags were impractical or of no use during work tasks that required close interaction:

If you already have the mindset to keep distance, the tag is not useful. Also, our team could not fully use them since we often worked closely together which made face masks the alternative.

Survey respondent

Good idea and it probably worked well for teams other than mine, but people became more aware in shared areas.

Survey respondent

It was also noted by several respondents that these types of devices can cause frustration when the physical setting or type of work does not allow for proper distance and the alarm goes off:

In some environments or during recordings and so forth, this noise can be disturbing. Sometimes it needs to be switched off when people need to be close or during recording. This is the only thing I see as potentially negative.

Interview C

It probably works well in situations where you are not dependent on staying close to others during work.

Survey respondent

Interviewees and survey respondents described many situations in which the distance tags needed to be switched off to avoid the alarm signal, which would disturb key activities such as recording sessions. A few individuals switched off the tag because they simply preferred to do so, indicating a need to check for adherence to safety recommendations.

In the survey, the participants were asked whether they were in the habit of switching off the distance tag in situations when it should have been used (Table 4).

The descriptive statistics show that a slight majority of the respondents, that is, 57% (17/30) of participants never or only seldom switched off the tag, while 12 did so sometimes or often and 1 person avoided using the tag as much as possible.

**Table 4:** Response distribution of how often the respondents switched off the tag in situations when it should have been used.

	Never—I attempted to use the tag as much as possible	I have done that very seldom	Sometimes	I often switched it off	I tried to avoid wearing the tag or have it switched on as much as possible
How often did you switch off the tag in situations when it should have been used?	13	4	6	6	1

#### Understanding and Learning

In its most basic function, the UWB-based distance tag is straightforward to use. It comes in the form of a card that hangs around a user’s neck. The interviewees stated that the tag provided an alarm, a high-pitched sound, whenever individuals got too close to other tag users. The sound stopped as soon as the tag users moved away from one another. This behavior did not require much instruction, and the purpose was easy to understand. As the app and contact-tracing functions were not used, the hardware device was the only interface that the users had to learn. The device did not require much from its user, except for keeping the device’s batteries charged and ensuring that they were turned on.

Table 5 shows that, in general, the survey respondents found it easy to learn how to use the tag: 90% (27/30) answered *agree* or *strongly agree*.

The distance tag could be switched off when the sound would have been inappropriate, but a downside of the switching-off function is that users sometimes forgot to switch it back on, as the interviewees described. The light indicating whether it was on or off was also confusing to some participants; it was red when it was active, which felt counterintuitive according to 1 survey respondent.

In addition to learning how to use the distance tag, there was also another learning process. The importance of understanding and learning how to maintain distance was described by multiple interviewees. They described a learning curve going through varied phases. In the beginning of the production, the participants had various attitudes, such as curiosity or skepticism. During the first week of use, the alarms went off frequently, which caused some frustration and reduced adherence. However, for some users, this was also a sign that they were not as good at maintaining distance as they thought:

I mean, from the beginning it was very strange, you started with “my God,” you stood a bit close, and it beeped. But then you learned to step back and then you started to appreciate it, because then you get it, you understand that it provides a function.

Interview A

After the informational campaign organized by the COVID-19 strategist, attitudes toward the tag and adherence to preventive use improved. The interviewees described that both communication and reminders were needed for these types of protective measures to work and to ensure that people used face masks and distance tags and washed their hands. Subsequently, habits started to form, and multiple interviewees described that the alarms sounded less frequently after a while. They explained that people learned to maintain a distance. At the end of the production, people were more meticulous about all the preventive measures. They expressed more appreciation and often asked for new tags. The general impression among the interviewees was that people were generally positive toward distance tags at the end of the production project and had learned how to act while wearing them.

There were also indications that this technology has the potential to facilitate habitual changes. Several interviewees stated that they are better or much better at maintaining their distance now, even when they were not wearing their devices:

My own experience is the best point of reference. You think about it, and even when you walk with a colleague to talk, you automatically keep the distance.

Interview C

I automatically move backwards now, even when I’m with friends.

Interview A

I’m more attentive to the behavior of others now. I wasn’t before. I thought of it as everyone’s own business. Now I react when someone gets too close. I’m so aware now, what 1.5 meters really means and what it can do.

Interview B

Table 6 shows the survey results concerning whether the tag was helpful when learning to maintain distance.

The overall results showed a slight majority of (57/90, 63%) *agree* and *strongly agree* answers, 24% (22/90) *disagree* or *strongly disagree* answers, and 12% (11/90) *neutral* answers*.*

**Table 5:** Ease of learning how to use the distance tags.

	Strongly disagree	Disagree	Neutral	Agree	Strongly agree
It was easy to learn how to use the tag	0	1	2	12	15

**Table 6:** The influence of distance tags while learning to maintain distance.

	Strongly disagree	Disagree	Neutral	Agree	Strongly agree
The tag helped me to keep distance	1	7	2	13	7
I am more aware of the distance even when I do not wear the tag	1	8	5	10	6
My colleagues became better at keeping distance due to the tag	0	5	4	14	7
Total	2	20	11	37	20

#### Accomplishing the Purpose

When the distance tags were first implemented, managerial staff had varying expectations. Some were skeptical and others were not sure what to expect or how the system would work. Some participants also expressed interest and curiosity. After a few weeks of using the distance tags, the general perception of the interviewees and most survey respondents was that the tags had been successful and fulfilled their purpose:

I was skeptical in the beginning but now I think this is a winning concept.

Interview B

I was a bit hesitant to how it would sound, how disruptive this would be. It showed to be something you adapted to very quickly and it is more helpful than disruptive.

Survey respondent

I didn’t know what to expect...Will this work?...Are they reliable?...But now I think it has worked great. They have absolutely fulfilled the function we thought it would.

Interview A

In addition to supporting physical distancing, the interviewees’ perceptions were that the distance tag helped safeguard the production because using these devices to monitor distance helped ensure safety on-site. The safety personnel said that the technology made sure that they “did not have to be everywhere all the time, to point out the distance” (Interview C). The tag took care of this basic control measure, and the tags were available everywhere. The interviewees expressed that the tags were a good complement to the safety personnel, who only had to ensure that people wore tags and that they had sufficient battery power. Furthermore, if not for the tags, the event would have required more security personnel to ensure that people maintained sufficient distance. However, 1 interviewee pointed out the following:

Adding more personnel is not always a solution during a pandemic. This would have added more potential sources for infection.

Interview C

Although interviewees expressed some concerns about the distance tags at the beginning of the production project, several of them experienced that the tags were necessary for maintaining physical distance at the workplace. One interviewee indicated that distance tags were essential for carrying out the event during the pandemic:

If we hadn’t used these devices, we wouldn’t have been able to go through with the event.

Interview B

Similar attitudes toward the positive effects of using the tags can be seen in the questionnaire answers (Table 7). The overall results showed of the 90 answers, a slight majority of *agree* and *strongly agree* answers 55 (61%), 14 (16%) *disagree* or *strongly disagree* answers, and 21 (23%) *neutral* answers.

The most negative comments related to the functionality of the device concerned battery life, which was deemed unsatisfactory. This point was mentioned by several respondents.

Individuals working at the event were advised to be careful, but they were not isolated from the outside world. Approximately 400 individuals were in place in total, when artists and their entourages were included. During the 6 weeks of the production project, 3 individuals in total were infected with the SARS-CoV-2 according to information from management. No further transmission was found in the workplace.

**Table 7:** Anticipated positive effects of using the distance tags.

	Strongly disagree	Disagree	Neutral	Agree	Strongly agree
As a whole, the tag is effective for changing individual behavior	0	3	3	17	7
I believe that the tag has helped us to reduce the spread of virus	0	3	10	12	5
Using the tag contributed to feeling safe and secure	1	7	8	13	1
Total	1	13	21	42	13

### Design Aspects

In this main theme, the focus is on the experiences of the distance tag’s functionality and interaction. Three subthemes are described in this section: *a purposefully annoying device*; *timing, tone, and proximity*; and *additional functions*.

#### A Purposefully Annoying Device

Several interviewees described that the device had a high-pitched noise, which caused irritation and frustration, especially in situations in which individuals were prevented from maintaining distance or in situations where the noise caused disruption:

[The device caused] a constant beeping in a large production...

Survey respondent

The device evoked various emotions among the participants. Some experienced frustration, while others felt that it provided safety by ensuring that everyone on-site maintained a sufficient distance:

It felt safe to use since it is easy to forget about the distance.

Survey respondent

I am incredibly positive! It has enabled us to be more attentive and, in that way, show respect for others.

Survey respondent

Table 8 shows how the survey respondents perceived the anticipated negative effects of using the distance tags.

The overall results showed that of the 90 answers, 19 (32%) *agree* and *strongly agree* answers, 35 (58%) *disagree* or *strongly disagree* answers, and 6 (10%) *neutral* answers*.* The respondents perceived that distance tags contributed to frustration more than they contributed to fatigue. Others commented that the alarm sound must be high pitched and annoying so that it fulfills the required effect, that is, so that people take immediate action:

I’m thinking that, it is good with a “bad” sound, because then you will move away.

Interview C

It is irritating but it needs to be.

Interview A

Had we had a more pleasant or a tone of increasing volume, we would not have reacted as quickly.

Interview B

Most of the survey respondents also found that the alarm signal served its purpose: 83% (25/30) answered *agree* or *strongly agree* (Table 9).

Table 10 shows whether survey respondents adapted to the audio signal by keeping sufficient distance or by ignoring the signal. Most of the respondents learned to keep safe distance: 76% (23/30) answered *agree* or *strongly agree*. No respondents were neutral about the suggestion that the alarm could be ignored: 46% (14/30) answered *agree* or *strongly agree* and 53% (16/30) answered *disagree* or *strongly disagree*.

**Table 8:** Anticipated negative effects of using the distance tags.

	Strongly disagree	Disagree	Neutral	Agree	Strongly agree
The tag contributed to frustration	3	8	2	14	3
The tag contributed to fatigue	18	6	4	2	0
Total	21	14	6	16	3

**Table 9:** How the audio signal was perceived.

	Strongly disagree	Disagree	Neutral	Agree	Strongly agree
The signal is intolerable, and it should be changed	10	9	7	1	3
The signal serves its purpose	2	1	2	12	13

**Table 10:** Survey respondents adapting to the audio signal.

	Strongly disagree	Disagree	Neutral	Agree	Strongly agree
Generally, it is easy to avoid the audio signal if you keep sufficient distance	0	5	2	14	9
I got used to the signal and could sometimes ignore it	10	6	0	11	3
Total	10	11	2	25	12

#### Timing, Tone, and Proximity

The distance tag gave off an alarm lasting a second or so after the distance was breached, for example, after passing another individual in a corridor. Some thought that this was disruptive, while others said that it was an effective way to know that the tag was active.

Table 11 shows opinions from the survey respondents concerning whether the audio signal should be activated when users pass each other or only if they stop too near each other. The result shows preference for the alarm sounding only if 2 individuals stop too close to each other. The option of having the alarm triggered even when individuals only passed each other got equally supported and opposed by the survey respondents.

Several interviewees and survey respondents asked for a vibration function that could be an alternative when an audio signal was not suitable:

[It works] fine, but it would be good if you could switch to a vibration on occasion such as sound recording.

Survey respondent

One interviewee said that she got used to the alarm and indicated a risk that one could start ignoring it after a while. It was proposed that the tone could change every time and avoid getting used to the tone. Others suggested that the tag could provide a prewarning, in the form of a softer sound or vibration, before the proximity is reached where the high-pitched alarm starts. Having a prealarm would let the user know that they were getting close to the threshold, and then they could potentially avoid the disturbing sound. However, most interviewees agreed that it was important for the device to be immediate when a certain proximity was reached. In addition, some respondents stated that the tone should neither be gradually increasing nor pleasant.

Table 12 shows the survey respondents’ opinions on 2 suggestions for the enhancement of the audio signal. A slight majority (17/30, 57%) of the respondents *strongly disagreed* or *disagreed* that a weaker signal would have the same effect, 23% (7/30) *strongly agreed* or *agreed* that a weaker signal would have the same effect, and 20% (6/30) were *neutral*. A total of 57% (17/30) of participants *agreed* or *strongly agreed* that there should be a prealarm warning, 30% (9/30) *disagreed* or *strongly disagreed*, and 13% (4/30) were neutral.

To fit the settings of the event, the event management set the distance for the tag alarm. The proximity detection was set to 150 cm, and the interviewees agreed that this was a good distance. The detection varied from +20 cm to −20 cm, which means that the alarm would sound from 130 cm to 170 cm, which seemed a reasonable distance in relation to the work tasks and the physical setting. At this alarm distance, most work activities were performed. According to interviewees, 2 full meters would have been too far. Interviewees experienced that the adjusted distance enabled people to talk to each other without raising their voices and they could socialize at the event, although with some distance:

*Now, we can have lunch together, but we sit with 1.5 meters apart, and we can be sure that it is exactly that distance. We don’t bring an extra chair, then the alarm would set off. But ithelps us to hang out, we can sit eye to eye and talk without a screen between us. I think it has assisted us in socializing.* [Interview B]

**Table 11:** When should the signal be heard?

	Strongly disagree	Disagree	Neutral	Agree	Strongly agree
It is appropriate that the signal is heard when we pass each other	4	9	4	9	4
It would be better if the signal is heard only if we stop close to each other, eg, after 2-3 seconds	5	3	3	13	6

**Table 12:** Suggestions for enhancement.

	Strongly disagree	Disagree	Neutral	Agree	Strongly agree
I believe that a weaker signal could have the same effect on my behavior	8	9	6	4	3
There should be a prior warning, eg, beep or vibration, before the audio signal starts	8	1	4	8	9
Total	16	10	10	12	12

#### Additional Functions

The interviewees and survey respondents agreed that the key function is the immediate warning provided by the distance tag. Some were interested in additional functions, such as data on the number of close interactions, or the use of these devices for contact tracing. Others wanted a battery charge indicator related to the previously mentioned complaints regarding the short battery time. Less than half of the survey respondents were interested in additional functions or in using an app connected to the device (12/30, 40% of respondents). However, attitudes differed and most clearly rejected those ideas (14/30, 46% of respondents) or were hesitant to add more features (3/30, 10% of respondents):

Absolutely NOT an app! But they could have a light indicating when the battery charge is becoming low.

Survey respondent

No, it should be uncomplicated. It would be good to see the percentage of the battery charge.

Survey respondent

No, I think it (tracking) would be a bit of an invasion of privacy, anyway, even if it had not been legislated. And I believe there is a side effect—in that you deprive the individual of their responsibility.

Interview B

The primary function is that they keep the distance...But I would have loved to use its full potential with all the functionality and using the app as well. Partly because it would be a fun thing, a gadget, it is making the product more interesting if you can check the app.

Interview C

It [tracking] could probably be something that would contribute to increased usage. Sounds like a smart idea for contact tracing.

Survey respondent

Many respondents were satisfied with the functions and did not wish for any additional tracking functions:

I think these are good. They provide us with freedom to move around. And they build on individual responsibility which is particularly important. We cannot organize in a way so that we constantly must check [the distance]. This is a very good support.

Interview B

### Summary of Findings

In total, 7 subthemes were constructed based on the qualitative analysis in this study. Key findings from the survey were added to these themes, and additional descriptive statistics are provided in Multimedia Appendix 1. The themes are summarized in Table 13, along with the key findings from both interviews (qualitative) and the survey (quantitative and qualitative).

**Table 13:** Themes, subthemes, and key findings of this study.

Theme, subtheme			Key finding
**Feasibility**			
	Implementation and acceptance—establishing a shared problem	Communication and clarifying the problem is important. Respondents reported that they had overestimated their capacity to maintain a sufficient distance. A station at the entrance can simplify the handout process. Attitudes vary and change over time; most users are positive afterward. A few are skeptical of the technology, but a majority support the intervention afterward.	
	Distance tags in context—strategy, environment, and activities	Distance tags are best suited as part of a larger preventive strategy. They are particularly useful in situations where individuals meet and tend to stand close by habit. Negative attitudes are often connected to situations where the tag is not useful or too disruptive for the situation. Usability depends on context and situation. Alternatives to the high-pitched alarm are needed for certain situations.	
	Understanding and learning	The distance tag was easy to use and understand. The indicator light was a little counterintuitive, and individuals sometimes forgot to switch the tag back on. It took a while to learn a new behavioral pattern; some expressed frustration in the beginning. There are indications that increased attention to distance remains even when the tag is removed.	
	Accomplishing the purpose	Most respondents agree that it supported physical distancing and behavior change. Users report more positive than negative effects. This technology is feasible when used in the right circumstances. A short battery life is the most negative aspect. Few got infected during the 6-week production project, indicating a preventive potential of the strategy as a whole.	
**Design aspects**			
	A purposefully annoying device	Multiple respondents agree that the high-pitched alarm is needed for immediate behavioral adaption—this should be the standard setting. A discrete nudge would not be sufficient in the long term. There are indications that users might get desensitized even to a high-pitched sound. The sound can cause frustration, but few people experience fatigue.	
	Timing, tone, and proximity	Timing and proximity were satisfactory. The tone fulfills its purpose, but alternatives are requested by some. Alternatives for certain situations can involve other measures, such as a switching-off function or vibration.	
	Additional functions	Some are interested in tracking functions and additional data, but it is not deemed necessary. A battery charge indicator is requested. Some are negative to tracking for privacy reasons. The direct warning enables individual responsibility, which is the most valuable function.	

## Discussion

### Principal Findings

A pandemic consists of a complex system of interacting factors, and many factors must be considered to prevent transmission. The behavior of individuals is a key issue in the transmission process [20] and was the focus of attention in the preventive strategy undertaken in this case. This study provides evidence for the rapidly emerging field of mobile preventive technology. Specifically, this study contributes to our understanding of the feasibility, use, and design of a specific wearable technology meant to maintain or improve physical distancing among its users.

Previous studies have reviewed or suggested a variety of designs that allow proximity detection and encourage physical distancing behavior [17-19,50]. However, few empirical studies have investigated the technical efficacy of proximity detection [19].

The objective of this study was to evaluate the feasibility of using a wearable device that nudges its user to maintain physical distance while also gathering information about how functionality and interactions with this device were experienced by the users. The following discussion focuses on the feasibility and design of distance tags.

### The Feasibility of Proximity-Based Technologies in a Pandemic Context

This case study illustrates that proximity-based nudging technologies are a feasible strategy for infectious disease management in a workplace where many individuals are in physical proximity and move around. Most users in this study supported the technology used in this intervention, would recommend it to others, agreed that it increased the user’s awareness of physical distancing, and believed that it was effective in changing individual behavior. A few were skeptical and at least 1 user was dissatisfied. Negative comments mostly concerned a poor fit between the technology and the work tasks or that there were situations in which physical distancing or a high-pitched audio signal were not appropriate. These comments highlight some opportunities for design (discussed in subsequent sections) and show that the usability of this technology is dependent on the context of use and the situation.

A founding idea behind nudging is to provide contextual feedback to facilitate better choices or behavior in distinctive situations [38]. Furthermore, previous studies have shown that context is important for understanding why and how nudges are effective [51]. In this case, the nudge is provided by a technology triggered by its user’s close interaction with others. For this nudge to be efficient, the physical context and activities must allow the user to either avoid or rapidly respond to the nudge. Therefore, before implementing this type of technology, it is recommended to undertake a systems thinking approach to understand how a nudge can be designed in relation to its context.

Systems thinking is the fundamental, conceptual pattern that makes it possible to ensure a holistic understanding of the situation [52]. This could involve modeling the problem and designing a preventive strategy as a system, defining the boundaries of the system in relation to the risk of transmission, the stakeholders involved and their perspectives, and their relations and activities in the context.

The choice of UWB instead of a Bluetooth device is in line with other recent studies on the efficacy of Bluetooth devices for contact-tracing purposes, indicating that proximity detection between individuals can be challenging in practice [53,54], and Kindt et al [54] specifically recommended UWB technology as an alternative to Bluetooth. However, as noted by Alo et al [50], UWB is more energy demanding than Bluetooth, and in this study, the battery life of the distance tags was deemed unsatisfactory.

The small number of individuals who became infected during this 6-week project is a possible indication that the overall preventive strategy—including handwashing, social bubbles, wearing face masks, and distance tags—was successful and that transmission in the workplace was minimized. To contextualize these findings, the “background” transmission of SARS-CoV-2 in Stockholm was among the highest in Sweden. At the time of this production project (weeks 5-13 in 2021), the number of infections in the city increased, peaking at 408 new cases per 100,000 individuals in week 13 [55]. This would indicate that approximately 10 new cases would be expected in a group of 400 persons during a 6-week period.

Workplaces have been important sites of transmission of COVID-19 [56], and the fact that this workplace can accommodate several individuals with a low amount of transmission is an encouraging sign. The workplace formed under this production project was not a closed system, meaning that individuals had contact with the outside world, even though they were advised to be careful. To put this in perspective, we also note that the activities undertaken in this case involved teamwork, physical labor, singing, and dancing, which theoretically may have put this workplace at a higher risk of widespread transmission. However, it is important not to draw conclusions about the preventive effects of individual preventive measures, as they were not studied separately. The combined effect of several preventive measures is an effective way to reduce transmission in public gatherings, and it is also likely that individuals that show adherence to one measure, such as consistent mask wearing, are practicing multiple measures [27]. Therefore, more studies are needed to confirm and ensure a sufficient preventive effect of nudging technologies to support preventive behavior.

### Design Aspects

A key finding regarding the design of distance tags is the users’ experience of the audio signal. The sound can be disruptive, but it did not cause fatigue. Generally, most respondents agreed that the audio signal needs to be annoying to have the intended effect.

Most users also indicated that their attention to distance and their long-term behavior were affected by wearing this device, an interesting finding that might be explained by the design and timing of the audio feature. The just-in-time prompt (the nudging feature) in this technology is in line with the Skinner operant conditioning [57,58], meaning that the high-pitched audio signal provides immediate feedback whenever a user is in close proximity to another user in a systematic and repetitive manner. The annoying signal is a form of positive punishment intended to minimize unwanted user behavior. The signal persists until the user returns, at which point the signal immediately stops. Withdrawal of unwanted signals is a form of negative reinforcement aimed at increasing the prevalence of distancing behavior. This audio feedback model can explain some users’ reports that they learned new and automated behavior after using the distance tag. Both approaches to behavioral design (nudging and operant conditioning) can be connected to behavioristic traditions that emphasize the effect of external stimuli on human behavior [57]. Specifically, we propose that nudging is useful in situations in which users fail to act in their best interests because of cognitive limitations, such as memory, attention span, or habits. Models, such as the Skinner operant conditioning, can provide more specific guidance for the design of a nudge’s interactive details, such as timing and pitch.

The other design aspects in this study concerned the types of feedback and information provided to users. For some users, having additional tracking functions and data on interactions could potentially be useful or motivational, although these features were not deemed necessary. At the same time, some users felt quite negatively about tracking and contact-tracing functions due to privacy and ethical concerns. The immediate warning provided sufficient support for this workplace to remain open during times of rising transmission in the region. The value of this technology was the opportunity for individuals to be able to act immediately and by this to be responsible for their own behavior. Compared with previous design proposals [17,18], the distance tag provided the same function, but in a more simplified design. By providing direct feedback and a clear mapping between the behavior and the feedback, it is straightforward and transparent.

There were indications in this study that some users may have become desensitized to the frequent alarm, even with a high-pitched tone. Thaler and Sunstein [38] noted that feedback for warning systems must be designed so that they do not provide warnings too frequently, because users will start to ignore them. The risk of desensitization is supported by previous research on other types of proximity systems used in the construction industry [30,31]. In this study, several participants asked for a vibration function instead of the audio alarm; at the same time, users clearly stated that too discrete a signal would be too easy to ignore. We can assume that a discrete notification used for longer durations would result in rapid desensitization, and the users would start to ignore the feedback entirely. We propose that a vibration should be available as a short-term alternative only when an audio signal is not appropriate or as a prealert to the audio signal.

It is possible that a warning system in 2 stages would be more advantageous, and we propose including a *dual nudge* in the design. In a dual nudge, the device would first vibrate, followed by high-pitched feedback if the situation is not corrected (in this case, if the user does not step back) a few seconds later. This idea is supported by previous research on warning systems in the automobile industry [35,37], which indicated that prealerts could help users gain control of the situation more quickly. Suzuki et al [35] suggested a combination alert, in which a vibration is followed by an audio signal: vibrations were found to be most appropriate for unpredictable conditions, whereas audio signals were best suited in predictable conditions. It has been shown that the response time to vibro-tactile signals is shorter than that of the other modes of alerts [31], which would also support the use of vibrations as the first step of a dual nudge. Furthermore, the user can more easily avoid the audio signal if a prewarning is delivered, which might alleviate some irritation. It may also improve the learning process if the user can quickly adapt to the behavior and avoid negative re-enforcement caused by the annoying sound. Less-frequent alarms would lower the risk of redundant alerts, alarm fatigue, and a desensitized user. The optimal timing of these 2 types of feedback (vibration and audio signal) and the relation to proximity needs to be further researched to optimize the speed of behavioral adaption as well as the user experience.

### Limitations

This study had several limitations. First, 8 interviews and 30 survey responses constituted a small sample size, which limits generalizability and prevents us from undertaking meaningful statistical comparisons within the sample group. Second, the interviews were conducted in the final week of the production and the survey a few weeks after the production project ended, so there may have been problems such as recall bias and the formulation of accurate recollections from the first weeks of production. Third, although the mixed methods and explorative approach enabled new and important insights into how and why things worked in practice, there were also fewer opportunities to make causal connections (compared with a controlled experiment). A major limitation of this study was the lack of a comparison group. In addition, any potential preventive effect of the distance tag cannot be distinguished from the effects of other preventive measures in place at the time, and it is likely that a combination of measures contributed to the observed low transmission rate. A final potential limitation is that this study was conducted in a Swedish context in a very specific workplace setting; even so, we think that our findings and conclusions may be applicable to other settings, for example, in other workplaces.

### Conclusions

This empirical study reports on the feasibility of using wearable technology to nudge individuals to maintain a safe distance in their workplace during a pandemic. The technology is particularly useful in places and situations where people meet and tend to stand close to each other, and it supports the attention to distance. The usability is dependent on the context and situation, which are crucial for the user’s ability to adapt. In situations where alarms are unavoidable or unsuitable, distance tags can be experienced as more frustrating than helpful. The study also demonstrated that this type of device is easy to understand and use, and it can be rapidly implemented with a handout station on-site. However, a learning curve needs to be considered in which the user gradually adapts to a new behavior, and users can expect more frequent alarms in the beginning. It is important that managers communicate and clarify the shared *problem* of physical distancing. Information and moral perspectives such as the need to protect others can facilitate acceptance and adherence. Most users agree that the audio signal needs to be irritating, and *purposefully annoying* feedback is suggested to be included in the design, to ensure sufficient behavioral adaption. Furthermore, we propose a *dual nudge* that involves a vibration followed by a sound to minimize the risk of desensitization. There are indications that the technology facilitates learning how to maintain a greater distance from others and that the behavior change can persist beyond the context of technology use.

This study concludes that nudging technologies based on proximity detection can be used to support this type of preventive behavior, focusing on maintaining physical distance from others. They facilitate physical distancing by providing a just-in-time prompt without the need for tracking contacts. This study provides insights into the emerging field of personal and wearable technologies used for primary preventive purposes during infectious disease outbreaks. Future research is needed to establish their preventive effects.

Furthermore, it would be interesting to explore the feasibility of this technology for outbreaks of other contagious diseases, particularly where transmission is dominated by close contact or respiratory droplets. Another avenue for future research is to investigate behavior change in more detail and in relation to the different forms of feedback provided by nudging technologies.

## Appendix

Multimedia Appendix 1 Survey questions.

Multimedia Appendix 2 Survey data closed questions.

## Abbreviations

CTA: contact-tracing app
UWB: ultrawide band

## Data Availability

All quantitative data generated and analyzed during this study are included in this published article and its supplementary information files. Owing to ethical considerations, the qualitative data generated and analyzed during this study are available from the corresponding author upon reasonable request.
