# Supplementary material for: Using a Proximity-Detection Technology to Nudge for Physical Distancing in a Swedish Workplace During the COVID-19 Pandemic: Retrospective Case Study
Source: JMIR Form Res. 2022 Dec 12;6(12):e39570. doi: 10.2196/39570 (PMC9746677; doi:10.2196/39570)
Supplement: Multimedia Appendix 1 [file formative_v6i12e39570_app1.docx]

## Multimedia Appendix 1

## Survey questions and answer alternatives

1. **Gender**

| **Alternatives** | **Corresponding value in the Excel file** |
| --- | --- |
| Woman | 1 |
| Man | 2 |
| Prefer not to answer | 3 |
| Other | 4 |

1. **Age (years)**

| Numeric value |
| --- |

1. **Work role**

| **Alternatives** | **Corresponding value in the Excel file** |
| --- | --- |
| Management/Personnel management | 1 |
| Security | 2 |
| Production | 3 |
| Artist/Entourage | 4 |
| Styling/Makeup | 5 |
| Other, please specify | 6 |

1. **How long did you use the distance tag?**

| **Alternatives** | **Corresponding value in the Excel file** |
| --- | --- |
| Less than a week | 1 |
| 1 week | 2 |
| 2 weeks | 3 |
| 3 weeks | 4 |
| 4 weeks | 5 |
| 5 weeks | 6 |
| 6 weeks | 7 |
| More than 6 weeks | 8 |

1. **What was your attitude towards using distance tags before the project?**

| **Alternatives** | **Corresponding value in the Excel file** |
| --- | --- |
| Negative – The tags will not work well | 1 |
| Sceptical | 2 |
| Neutral – Don’t know | 3 |
| Quite positive | 4 |
| Positive – The tags will work well | 5 |

1. **What is your attitude towards using distance tags now (after the project)?**

| **Alternatives** | **Corresponding value in the Excel file** |
| --- | --- |
| Negative – The tags did not work well | 1 |
| Sceptical | 2 |
| Neutral – Don’t know | 3 |
| Quite positive | 4 |
| Positive – The tags worked well | 5 |

1. **Comments?**

Open-ended question

1. **What kind of factors, for instance a specific kind of information, motivated you to use/not to use the distance tag?**

Open-ended question

1. **How often did you switch off the tag in situations when it should have been used?**

| **Alternatives** | **Corresponding value in the Excel file** |
| --- | --- |
| Never – I attempted to use the tag as much as possible when it was required | 1 |
| I have done that very seldom | 2 |
| Sometimes | 3 |
| I often switched it off | 4 |
| I tried to avoid wearing the tag or have it switched on as much as possible | 5 |

1. **Were you aware of the physical distancing and accustomed to keeping distance before you started using the distance tag?**

Open-ended question

1. **The tag helped me to keep distance.**

| **Alternatives** | **Corresponding value in the Excel file** |
| --- | --- |
| Strongly disagree | 1 |
| Disagree | 2 |
| Neutral/Don’t know | 3 |
| Agree | 4 |
| Strongly agree | 5 |

1. **I am more aware of the distance even when I don’t wear the tag.**

| **Alternatives** | **Corresponding value in the Excel file** |
| --- | --- |
| Strongly disagree | 1 |
| Disagree | 2 |
| Neutral/Don’t know | 3 |
| Agree | 4 |
| Strongly agree | 5 |

1. **My colleagues became better at keeping distance due to the tag.**

| **Alternatives** | **Corresponding value in the Excel file** |
| --- | --- |
| Strongly disagree | 1 |
| Disagree | 2 |
| Neutral/Don’t know | 3 |
| Agree | 4 |
| Strongly agree | 5 |

1. **As a whole, the tag is effective for changing individual behaviour.**

| **Alternatives** | **Corresponding value in the Excel file** |
| --- | --- |
| Strongly disagree | 1 |
| Disagree | 2 |
| Neutral/Don’t know | 3 |
| Agree | 4 |
| Strongly agree | 5 |

1. **I believe that the tag has helped us to reduce the spread of virus**

| **Alternatives** | **Corresponding value in the Excel file** |
| --- | --- |
| Strongly disagree | 1 |
| Disagree | 2 |
| Neutral/Don’t know | 3 |
| Agree | 4 |
| Strongly agree | 5 |

1. **It was easy to learn how to use the tag.**

| **Alternatives** | **Corresponding value in the Excel file** |
| --- | --- |
| Strongly disagree | 1 |
| Disagree | 2 |
| Neutral/Don’t know | 3 |
| Agree | 4 |
| Strongly agree | 5 |

1. **The tag contributed to frustration.**

| **Alternatives** | **Corresponding value in the Excel file** |
| --- | --- |
| Strongly disagree | 1 |
| Disagree | 2 |
| Neutral/Don’t know | 3 |
| Agree | 4 |
| Strongly agree | 5 |

1. **The tag contributed to fatigue.**

| **Alternatives** | **Corresponding value in the Excel file** |
| --- | --- |
| Strongly disagree | 1 |
| Disagree | 2 |
| Neutral/Don’t know | 3 |
| Agree | 4 |
| Strongly agree | 5 |

1. **Using the tag contributed to feeling safe and secure.**

| **Alternatives** | **Corresponding value in the Excel file** |
| --- | --- |
| Strongly disagree | 1 |
| Disagree | 2 |
| Neutral/Don’t know | 3 |
| Agree | 4 |
| Strongly agree | 5 |

1. **As a whole, I support this effort.**

| **Alternatives** | **Corresponding value in the Excel file** |
| --- | --- |
| Strongly disagree | 1 |
| Disagree | 2 |
| Neutral/Don’t know | 3 |
| Agree | 4 |
| Strongly agree | 5 |

1. **I ‘d recommend the tag for other workplaces.**

| **Alternatives** | **Corresponding value in the Excel file** |
| --- | --- |
| Strongly disagree | 1 |
| Disagree | 2 |
| Neutral/Don’t know | 3 |
| Agree | 4 |
| Strongly agree | 5 |

1. **What is your overall experience of using the distance tag?**

Open-ended question

1. **Generally, it is easy to avoid the audio signal if you keep sufficient distance.**

| **Alternatives** | **Corresponding value in the Excel file** |
| --- | --- |
| Strongly disagree | 1 |
| Disagree | 2 |
| Neutral/Don’t know | 3 |
| Agree | 4 |
| Strongly agree | 5 |

1. **The signal serves its purpose.**

| **Alternatives** | **Corresponding value in the Excel file** |
| --- | --- |
| Strongly disagree | 1 |
| Disagree | 2 |
| Neutral/Don’t know | 3 |
| Agree | 4 |
| Strongly agree | 5 |

1. **I believe that a weaker signal could have the same effect on my behaviour.**

| **Alternatives** | **Corresponding value in the Excel file** |
| --- | --- |
| Strongly disagree | 1 |
| Disagree | 2 |
| Neutral/Don’t know | 3 |
| Agree | 4 |
| Strongly agree | 5 |

1. **The signal is intolerable, and it should be changed.**

| **Alternatives** | **Corresponding value in the Excel file** |
| --- | --- |
| Strongly disagree | 1 |
| Disagree | 2 |
| Neutral/Don’t know | 3 |
| Agree | 4 |
| Strongly agree | 5 |

1. **I got used to the signal and could sometimes ignore it.**

| **Alternatives** | **Corresponding value in the Excel file** |
| --- | --- |
| Strongly disagree | 1 |
| Disagree | 2 |
| Neutral/Don’t know | 3 |
| Agree | 4 |
| Strongly agree | 5 |

1. **There should be a prior warning, e.g., beep or vibration, before the audio signal starts.**

| **Alternatives** | **Corresponding value in the Excel file** |
| --- | --- |
| Strongly disagree | 1 |
| Disagree | 2 |
| Neutral/Don’t know | 3 |
| Agree | 4 |
| Strongly agree | 5 |

1. **It is appropriate that the signal is heard when we pass each other.**

| **Alternatives** | **Corresponding value in the Excel file** |
| --- | --- |
| Strongly disagree | 1 |
| Disagree | 2 |
| Neutral/Don’t know | 3 |
| Agree | 4 |
| Strongly agree | 5 |

1. **It would be better if the signal is heard only if we stop close to each other, e.g., after 2-3 seconds.**

| **Alternatives** | **Corresponding value in the Excel file** |
| --- | --- |
| Strongly disagree | 1 |
| Disagree | 2 |
| Neutral/Don’t know | 3 |
| Agree | 4 |
| Strongly agree | 5 |

1. **What is your overall experience of how the distance tag communicates with its user?**

Open-ended question

1. **Would you like to have an app connected to the distance tag, or have access to more information or statistics?**

Open-ended question

1. **What worked well; what would be the most important success factors of using the distance tag?**

Open-ended question

1. **What did not work well when using the distance tag; were there any superfluous functions?**

Open-ended question

1. **Any other thoughts, requirements or ideas concerning this topic? Are mobile technologies capable to support you in other ways during the pandemic?**

Open-ended question
